# Efficacy of Risankizumab in Moderate to Severe Psoriasis Considering Body Mass Index: A Scoping Review

**DOI:** 10.7759/cureus.105055

**Published:** 2026-03-11

**Authors:** Karina Sarmiento, Mariana Siqueiros, Maximiliano Hernández, Gerardo Morales, Victor Orozco, Luis F Pumarejo, Aldo D Gonzalez

**Affiliations:** 1 Medicine, Universidad Autónoma de Guadalajara, Guadalajara, MEX; 2 Public Health, Hospitales Civiles de Guadalajara, Guadalajara, MEX; 3 Medicine, Universidad Autónoma De Guadalajara, Guadalajara, MEX

**Keywords:** dqli, obesity, pasi, psoriasis, risankizumab

## Abstract

Psoriasis is a chronic, immune-mediated inflammatory skin disease that significantly impacts patients quality of life and is frequently associated with obesity. This comorbidity may influence disease progression and treatment outcomes. Risankizumab, an interleukin-23 (IL-23) inhibitor, has demonstrated high efficacy in treating moderate to severe plaque psoriasis. However, its performance in obese patients remains unclear due to conflicting data.

This scoping review aims to evaluate the impact of obesity, specifically BMI ≥ 30 kg/m², on the efficacy and safety of risankizumab in adults with moderate to severe plaque psoriasis.

A comprehensive literature search was carried out in databases including PubMed, Scopus, JAMA, Elsevier, and Wiley, between December 2024 and February 2025. The selection included studies from 2020 to 2025, focusing on Psoriasis Area and Severity Index (PASI) and Dermatology Life Quality Index (DLQI) outcomes in psoriatic patients receiving risankizumab, with or without obesity. 24 articles were included and analyzed by study design, patient characteristics, treatment response, and BMI influence.

Risankizumab consistently demonstrated strong efficacy and safety in reducing PASI scores and improving DLQI across all BMI categories. While most studies reported no significant difference in response due to BMI, some suggested enhanced early responses in non-obese patients. Continuous treatment showed long-term benefits regardless of BMI, with 90% or greater improvement in the PASI (PASI90) and complete clearance (PASI100) maintained up to five years.

Risankizumab is a highly effective and safe biologic therapy for moderate to severe psoriasis, even in patients across all BMI. Although some variations in response exist, the overall evidence supports its use across diverse patient profiles. Weight management should still be considered a complementary strategy in psoriasis care.

## Introduction and background

Psoriasis is a chronic, immune-mediated inflammatory skin disease that affects approximately 3% of the global population [1]. Characterized by the formation of thick, scaly plaques, psoriasis not only impairs physical appearance but also significantly diminishes patients’ quality of life, often leading to psychological and social challenges. It poses a significant public health burden due to its impact on quality of life and its strong association with various comorbidities. Among these, obesity has emerged as one of the most relevant factors, given its bidirectional relationship with psoriasis. Obesity not only increases the risk of developing psoriasis but also worsens disease severity and treatment response, likely due to the persistent state of systemic inflammation present in both conditions. [2]

Multiple treatment options are available for patients with moderate to severe plaque psoriasis including phototherapy, steroidal and nonsteroidal topical agents, and systemic nonbiologic and biologic therapies. In recent years, biologic therapies have revolutionized the treatment landscape for moderate to severe psoriasis. Risankizumab, is a humanized immunoglobulin G1 (IgG1) antibody that binds to the p19 subunit and inhibits interleukin-23 (IL-23) risankizumab has demonstrated remarkable efficacy in reducing disease severity and improving patients’ quality of life [1]. However, questions remain regarding its effectiveness and safety in obese patients, as body weight and adiposity may influence drug pharmacokinetics and immune responses.

Multiple clinical assessment methods are used to evaluate disease burden, with the most relevant being the Psoriasis Area and Severity Index (PASI). Similarly, various patient-reported outcomes are commonly used, such as the Dermatology Life Quality Index (DLQI), as well as measures of fatigue, skin pain, and itching [1].

To date, the scientific literature on the influence of BMI on risankizumab efficacy is still limited and, in some cases, contradictory. Given these discrepancies, a systematic analysis of the available evidence is crucial to better inform clinical decision-making.

The primary aim of this review is to evaluate the impact of obesity on the efficacy and safety of risankizumab in patients with moderate to severe plaque psoriasis. Specifically, the study seeks to analyze clinical responses in obese individuals, assess the influence of BMI on treatment durability, and identify potential biological mechanisms underlying these variations. Ultimately, this research intends to contribute relevant evidence to support the development of personalized treatment strategies and improve clinical outcomes for this patient population.

Among the many comorbidities associated with psoriasis, obesity has gained increasing recognition as a significant factor influencing disease severity and treatment outcomes. The bidirectional relationship between psoriasis and obesity suggests that each condition can exacerbate the other. Obesity, characterized by excess adipose tissue, contributes to a persistent state of systemic inflammation, which can intensify psoriasis symptoms and reduce responsiveness to therapy. Conversely, psoriasis-related inflammation may promote metabolic disturbances that favor weight gain. This interplay complicates clinical management, as patients with higher BMI often exhibit more severe disease and may respond less favorably to conventional treatments [2].

The advent of biologic therapies has revolutionized the management of moderate to severe psoriasis, offering high efficacy and improved safety profiles compared to traditional systemic treatments. Risankizumab, a humanized IgG1 monoclonal antibody targeting the p19 subunit of IL-23, stands out among these agents due to its potent anti-inflammatory effects. Clinical trials have demonstrated that risankizumab effectively reduces psoriasis severity, enhances skin clearance, and improves patient-reported outcomes such as quality of life [3]. Nevertheless, a critical question persists regarding its performance in obese patients. Since pharmacokinetics and immune responses can be influenced by adiposity, it is imperative to understand whether body weight impacts the drug’s efficacy and safety.

The impact of obesity on the therapeutic response to risankizumab remains a subject of discussion. Higher BMI has been proposed as a potential factor associated with reduced treatment response, possibly related to altered drug distribution or immune modulation. Conversely, comparable clinical improvements and sustained long‑term responses have also been observed across different BMI categories.

The objective of this scoping review is to systematically assess whether obesity, defined as a BMI ≥ 30 kg/m², reduces the therapeutic effectiveness of risankizumab in adults with moderate to severe plaque psoriasis.

By analyzing studies published between 2020 and 2025, the scoping review seeks to clarify whether weight influences key outcomes such as the PASI response rates and improvements in quality of life measures like the DLQI. Additionally, it explores potential biological mechanisms underlying differences in treatment response, considering factors such as drug pharmacokinetics and systemic inflammation.

The ultimate goal is to provide clinicians with evidence-based insights that support personalized treatment strategies, especially for patients with obesity - a group at higher risk for severe disease and poorer outcomes. Recognizing the importance of weight management as a complementary approach, this scoping review underscores the necessity of integrating pharmacologic and lifestyle interventions to optimize clinical outcomes. As psoriasis patients with elevated BMI continue to grow in number, understanding how biologic therapies like risankizumab perform in this subgroup will be critical for improving overall disease management and enhancing patients’ quality of life.

A scoping review was conducted inspired by the methodological frameworks of Arksey et al. and Levac et al. [4,5]. The research question was developed through group discussion and refined to target a specific population (adult patients with moderate to severe plaque psoriasis), a concept (risankizumab), and relevant clinical outcomes (PASI and DLQI). Although obesity (defined as BMI ≥ 30 kg/m²) was considered a clinically relevant characteristic, it was not included as an inclusion criteria.

An iterative process was used to identify appropriate keywords and synonyms in English and Spanish (Table 1), including terms such as “psoriasis,” “risankizumab,” “PASI,” “DLQI,” “obesity,” “BMI,” “comorbidities,” and “safety,” applying Boolean operators (“AND,” “OR,” “NOT”) with database‑specific adaptations. For example, the PubMed search strategy included (“psoriasis” AND “risankizumab”) AND (“PASI” OR “DLQI”) AND (“obesity” OR “BMI”). The search was conducted in PubMed, Scopus, and Google Scholar for studies published between January 2020 and September 2025.

**Table 1:** Keywords in the scoping review about the efficacy of risankizumab in moderate to severe psoriasis considering BMI PASI: Psoriasis Area and Severity Index; DLQI: Dermatology Life Quality Index; IL: Interleukin

Clinical Condition	Therapeutic Intervention	Participant Characteristics	Outcome Measures
Psoriasis	Rizankizumab	Obesity	PASI
Plaque psoriasis	Clinical trial	High BMI	DQLI
Inflammatory skin disease	IL-23 inhibitor	-	PASI
Chronic psoriasis	Biologic therapy	-	DLQI

Studies were eligible if they enrolled adult patients with moderate to severe plaque psoriasis, were published in English or Spanish, and reported at least one relevant clinical outcome (PASI75, PASI90, PASI100, or DLQI). BMI data were not required for inclusion but were recorded when available. The exclusion criteria were applied when the research did not evaluate risankizumab, included pediatric populations, was published in languages other than English or Spanish, or lacked relevant clinical outcomes related to efficacy, quality of life, or safety. The research was limited to publications from 2020 because risankizumab was first approved in 2019; older studies would not provide relevant data on its efficacy or safety.

Data were charted using a standardized extraction form that captured study design, population characteristics, sample size, baseline disease severity, outcomes (PASI, DLQI), safety findings, and, when available, BMI or obesity status, with extraction performed independently by two reviewers to ensure consistency. Extracted data were synthesized using a descriptive and narrative approach to summarize trends in clinical efficacy, patient‑reported outcomes, and safety across studies; when obesity data were available, results were compared descriptively between obese and non‑obese subgroups, without conducting a meta‑analysis due to heterogeneity in study designs and reporting.

**Figure 1:**
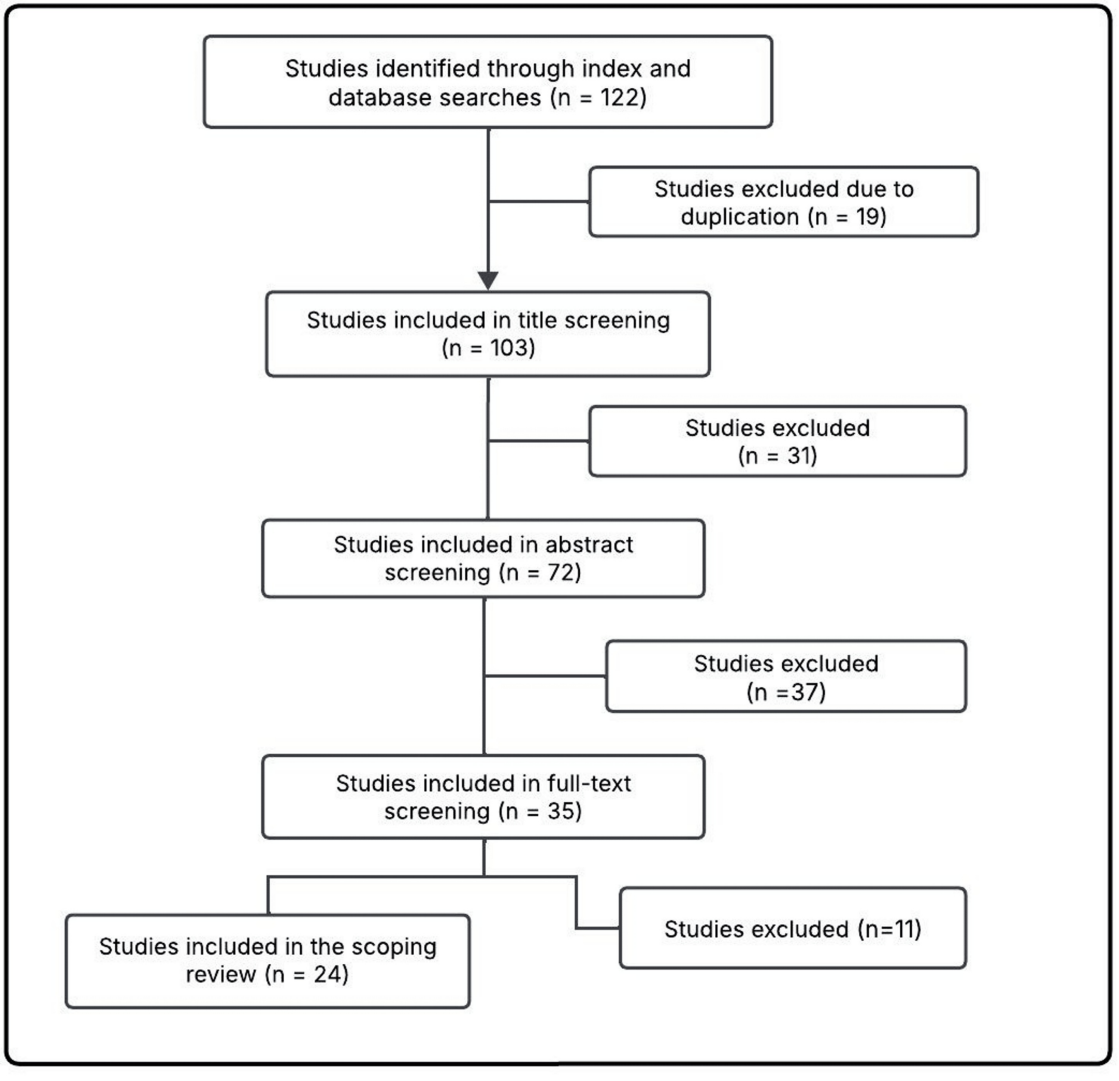
Selection process of the studies in the scoping review of the effectiveness of risankizumab in moderate to severe psoriasis considering BMI: 2020-2025

The screening process followed Preferred Reporting Items for Systematic Reviews and Meta-Analyses (PRISMA) guidelines (Figure 1). Duplicates were removed, and titles and abstracts were screened independently by four reviewers. Any discrepancies were resolved through discussion and consensus among all authors.

Full texts of potentially eligible studies were assessed, and 24 studies met the inclusion criteria. These were synthesized in an evidence matrix summarizing the author, objective, methodology, and main results (Table 2). To further enhance the interpretability of the findings, a comparative summary of the included studies is provided (Table 3), presenting the main efficacy outcomes, quality-of-life improvements, and the influence of BMI on therapeutic response.

**Table 2:** Included studies for the scoping review of the effectiveness of risankizumab in moderate to severe psoriasis considering BMI, 2020-2025

Study	Country	Methodology	Therapeutic Function of Risankizumab	Efficacy of Risankizumab Against Psoriasis	Relationship between Obesity and Psoriasis	Efficacy of Risankizumab in Patients with Moderate to Severe Plaque Psoriasis Based on BMI
Strober et al. [1]	USA	Retrospective observational cohort study	X			
Czarnecka et al. [2]	Poland	Observational cohort study			X	
Gooderham et al. [3]	France	Randomized clinical trial		X		
Crowley et al. [6]	USA	Prospective randomized controlled clinical trial	X			X
Strober et al. [7]	USA	Prospective randomized controlled clinical trial	X	X		
Adamcyzk et al. [8]	Poland	Multicenter retrospective study	X			
Gordon et al. [9]	USA	Controlled clinical trial	X			
Martorell-Calatayud et al. [10]	Spain	Retrospective cohort study	X			X
Agencia Española de Medicamentos [11]	Spain	Randomized clinical trial	X			
Borroni et al. [12]	Italy	Case control retrospective study		X		
Blauvelt et al. [13]	USA	Randomized clinical trial		X		
Megna et al. [14]	Italy	Retrospective cohort study		X		
Papp et al. [15]	USA	Longitudinal cohort study		X		X
Garguilo et al. [16]	Italy	Retrospective cohort study		X		
Timis et al. [17]	Romania	Prospective cohort study			X	
Rompoti et al. [18]	Greece	Retrospective cohort study			X	
Caldarola et al. [19]	Italy	Retrospective cohort study	X			
Vaiopoulos et al. [20]	Greece	Retrospective observational cohort study				X
Gargiulo et al. [21]	Italy	Retrospective cohort study				X
Ibba et al. [22]	Italy	Retrospective cohort study				X
Tejedor et al. [23]	Spain	Retrospective observational cohort study				X
Mastorino et al. [24]	Italy	Retrospective observational cohort study		X		
Östör et al. [25]	UK	Randomized controlled clinical trial		X		
Warren et al. [26]	UK	Non-randomized prospective experimental before and after study	X			X

**Table 3:** Comparative analysis of the included studies evaluating risankizumab efficacy considering BMI IL: Interleukin; PASI: Psoriasis Area and Severity Index; DLQI: Dermatology Life Quality Index

Study	Country	Study Design	Sample Size (n)	BMI Impact	PASI 90 (%)	PASI 100 (%)	DLQI Improvement	Key Findings
Strober et al. [1]	USA	Retrospective observational cohort study	287	No significant difference by BMI	66	50	Yes	High skin clearance and significant DLQI improvement after 12 months of risankizumab treatment
Czarnecka et al. [2]	Poland	Observational cohort study	147	Higher BMI associated with greater psoriasis severity	No reported	No reported	No reported	Overweight and obesity were significant clinical factors correlated with increased psoriasis severity
Gooderham et al. [3]	France	Randomized controlled trial	1600	Comparable efficacy across BMI categories	80-85	55.60	Yes	Risankizumab showed durable long‑term PASI and quality‑of‑life improvements regardless of BMI
Crowley et al. [6]	USA	Prospective randomized controlled clinical trial	120	None	88	54	Yes	BMI and weight did not significantly affect outcomes
Strober et al. [7]	USA	Randomized controlled trial	506	No difference by BMI	84	52	Yes	Comparable efficacy across BMI <25, 25-30, and ≥30 kg/m²
Adamczyk et al. [8]	Poland	Multicenter retrospective study	185	Effective across BMI categories	80-85	55-60	Yes	Risankizumab showed high effectiveness and sustained response in real‑life patients with moderate to severe psoriasis
Gordon et al. [9]	USA	Controlled clinical trial	3000	Not analyzed	No reported	No reported	No reported	Long‑term analysis showed a favorable and consistent safety profile of risankizumab in patients with psoriatic disease
Martorell-Calatayud et al. [10]	Spain	Retrospective cohort	180	Mild reduction in BMI ≥30 kg/m²	85	67	Yes	Non-obese achieved faster early PASI ≤ 2 responses
Agencia Española de Medicamentos [11]	Spain	Therapeutic positioning report	Not applicable	Not analyzed	75	35-40	Yes	Risankizumab shows high efficacy and favorable safety in moderate to severe plaque psoriasis according to pivotal clinical trial evidence
Borroni et al. [12]	Italy	Case-controlled retrospective study	166	Effective regardless of BMI	80-85	55-60	Yes	Risankizumab showed high real‑world effectiveness and a favorable safety profile in patients with moderate to severe plaque psoriasis after 40 weeks of treatment
Blauvelt et al. [13]	USA	Randomized clinical trial	507	Not analyzed	90	80	Yes	Continuous therapy showed durable skin clearance at 52-104 weeks
Megna et al. [14]	Italy	Retrospective cohort study	112	Effective across BMI categories	85-90	60-65	Yes	Long‑term real‑life treatment with risankizumab maintained high efficacy and a favorable safety profile over 2 years in patients with moderate to severe psoriasis
Papp et al. [15]	USA	Longitudinal cohort study	897	Not analyzed directly	85	52	Yes	Maintained efficacy up to 5 years regardless of BMI
Gargiulo et al. [16]	Italy	Retrospective cohort study	95	No impact after week 52	90	65	Yes	Sustained efficacy regardless of BMI
Timis et al. [17]	Romania	Prospective cohort study	70	Not analyzed	No reported	No reported	No reported	Biologic therapy in psoriasis patients was associated with reduced systemic inflammation and changes in metabolic syndrome parameters during follow‑up
Rompoti et al. [18]	Greece	Retrospective cohort study	112	Obesity common (>35%)	82	60	Yes	Obesity correlated with disease severity but not response to risankizumab
Caldarola et al. [19]	Italy	Retrospective cohort study	100	None	86	61	Yes	BMI did not affect clinical outcomes after 1 year of treatment
Vaiopolus et al. [20]	Greece	Retrospective observational cohort study	86	Not specifically evaluated	No reported	No reported	No reported	IL‑23 inhibitors showed high real‑world efficacy in psoriatic arthritis and difficult‑to‑treat psoriasis areas
Gargiulo et al. [21]	Italy	Retrospective cohort study	131	Not specifically evaluated	82-85	55-60	Yes	Risankizumab demonstrated high real‑world effectiveness and a favorable safety profile in moderate to severe plaque psoriasis over 52 weeks
Ibba et al. [22]	Italy	Retrospective cohort	70	Slightly lower in obese	80	63	Yes	Comparable long-term efficacy in patients with cardiometabolic disease
Tejedor et al. [23]	Spain	Retrospective observational cohort study	84	Not stratified, high BMI prevalence	71	57	Yes	Maintained response despite metabolic comorbidities
Mastorino et al. [24]	Italy	Retrospective observational cohort study	166	Not specifically evaluated	84	60	Yes	Sustained high efficacy of risankizumab through week 52
Östör et al. [25]	UK	Randomized controlled clinical trial	443	Not specifically analyzed	Not reported	Not reported	Yes	Risankizumab improved outcomes in active psoriatic arthritis at week 24 with good safety
Warren et al. [26]	UK	Non-randomized prospective experimental before and after study	252	Not evaluated	66	40	Yes	Switching to risankizumab led to substantial clinical improvement in psoriasis patients with inadequate response to IL‑17 inhibitors

**Figure 2:**
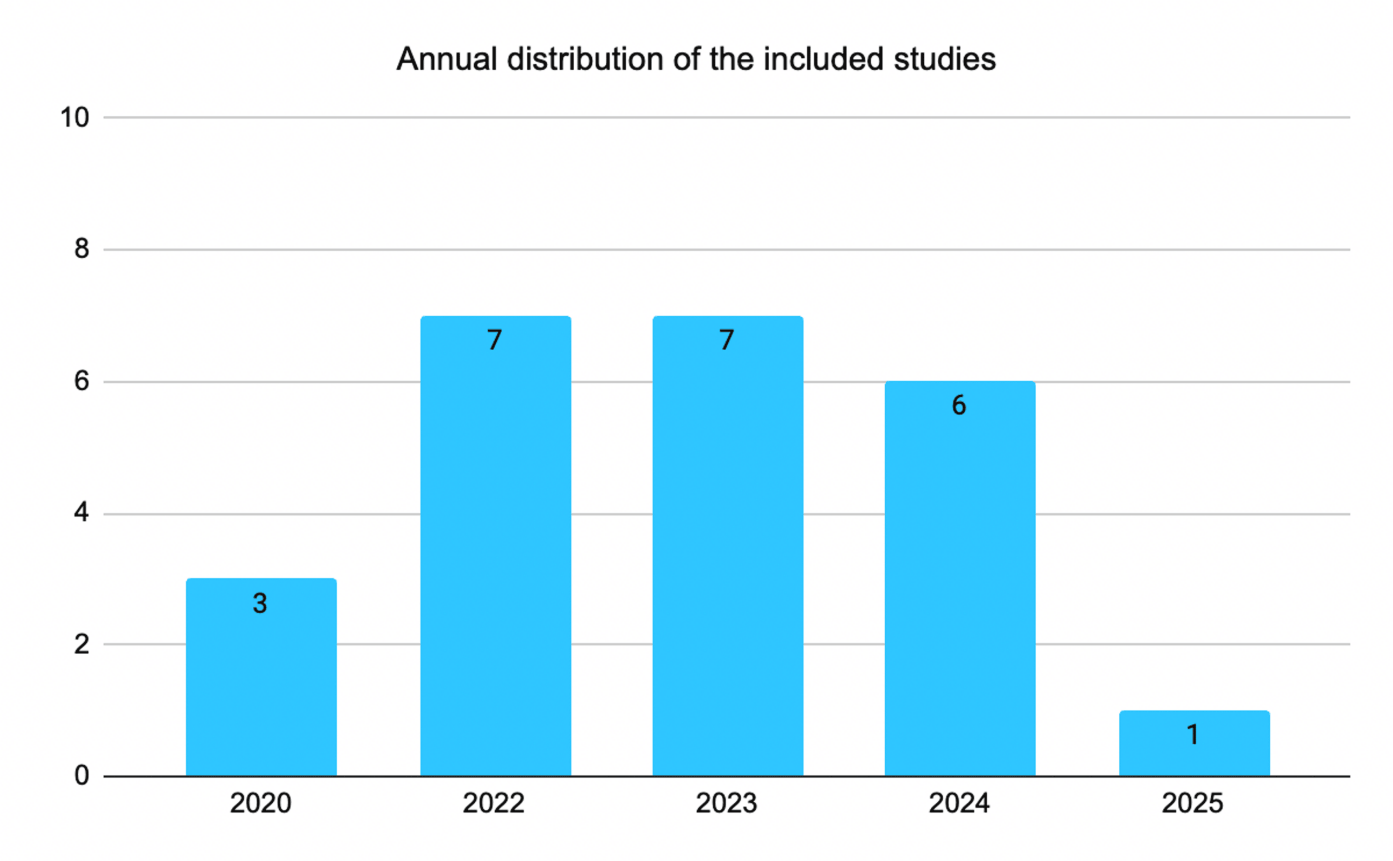
Annual distribution of the included studies of the scoping review of the effectiveness of risankizumab in moderate to severe psoriasis considering BMI, 2020-2025

In addition to efficacy outcomes, the matrix also described methodological features, study region, and how each study addressed therapeutic function or the association between obesity and psoriasis. Figure 2 shows the temporal distribution of the included studies, highlighting the emerging nature of this literature.

## Review

Therapeutic function of risankizumab

The results indicate a significant improvement in disease severity, with a high response rate in terms of PASI reduction. In terms of safety, the drug maintained a favorable profile, with a low incidence of serious adverse events [6]. In contrast, those who discontinued treatment experienced a higher relapse rate, highlighting the importance of continued use of risankizumab for effective and safe psoriasis control [7].

All patients with similar disease characteristics were evaluated randomized with risankizumab and ustekinumab, in which risankizumab showed to be more effective at weeks 16 and 52 achieving a PASI of 90, proving that the safety profile of risankizumab is in agreement with previously published data [8].

A total of 444 patients were randomized to receive risankizumab (n = 224) or placebo (n = 220). After 24 weeks, patients treated with risankizumab showed a significantly greater improvement in symptoms compared with placebo (51.3% vs. 26.5%). In addition, risankizumab was associated with a lower incidence of adverse events than placebo (0.9% vs. 2.3%) [9].

It mentions the long-term safety of risankizumab in patients with psoriasis and psoriatic arthritis, and notes that adverse effects were similar in both groups [10].

In the clinical studies reviewed, risankizumab (Skyrizi®) demonstrated superior efficacy in skin clearance compared to other biologics such as ustekinumab, adalimumab and secukinumab achieving a higher proportion of patients with a 90% or greater improvement in the PASI (PASI90), indicating a 90% reduction in psoriasis severity. In addition, a significant improvement in quality of life was observed among treated patients. The short-term safety profile was favorable, although long-term monitoring is warranted. These findings support its positioning as a preferred therapeutic alternative in patients eligible for systemic treatment [1,11]. 

Efficacy of risankizumab against psoriasis

Given its central role in the pathogenesis of chronic plaque psoriasis, IL-23 - particularly its p19 subunit - has emerged as a key therapeutic target. Risankizumab, a humanized monoclonal antibody that selectively inhibits the p19 subunit of IL-23, disrupts the IL-23/Th17 axis responsible for sustained inflammation and keratinocyte hyperproliferation [12].

According to Gooderham et al., risankizumab achieved significant improvements in both PASI and DLQI scores [11]. A greater proportion of patients on risankizumab reached PASI ≤3, PASI ≤1, and PASI = 0 compared to those treated with ustekinumab. Furthermore, risankizumab demonstrated a faster time to reach these efficacy thresholds. In long-term follow-up (up to 172 weeks), more than 90% of patients sustained PASI ≤3, and over 80% achieved DLQI 0/1, reflecting durable and significant improvements in both skin condition and quality of life [5].

Blauvelt et al. pointed out that patients receiving continuous risankizumab therapy experienced sustained and high rates of PASI90 and PASI100 responses at weeks 52 and 104. In contrast, patients who had treatment withdrawn showed a notable decline in their response [13].

Megna et al. confirm the efficacy and safety of risankizumab up to 104 weeks of treatment [14]. Papp et al. mention that with 256 weeks of treatment, 85.1% of patients achieved PASI90, while 52.3% achieved PASI100 [15]. These results indicate that continuous treatment with risankizumab for up to five years maintains high and sustained efficacy in patients with moderate to severe plaque psoriasis [14,15].

Gargiulo et al. add that after three years, complete or almost complete skin clearance was observed in all patients with the involvement of scalp, genitalia, and palms/soles, while 95% of patients with onychopathy achieved the same outcome [16]. Strober et al. notes that patients taking risankizumab achieved statistically greater mean improvement in PASI scores at week 52 compared with patients receiving ustekinumabby BMI (<25, 25-<30, ≥30), weight (≤100 kg or >100 kg) and weight quartile [7].

Relationship Between Obesity and Psoriasis

The association between psoriasis and obesity is well-documented, with multiple studies highlighting a strong predisposition for abnormal body mass in psoriatic patients. In an observational cohort study, Czarnecka et al. reported that overweightness (39.46%) and obesity (37.41%) were highly prevalent, emphasizing the increased risk of weight-related complications in this population [2].

Similarly, findings from a prospective cohort study by Timis et al. revealed that 41.51% of cases presented with obesity, further reinforcing the high burden of excess weight in psoriatic individuals. These results collectively support the notion that obesity is a frequent comorbidity in psoriasis, which may have implications for disease progression and management strategies [17].

In an observational cohort study, Czarnecka et al. demonstrated that overweightness and obesity significantly influenced psoriasis severity, as indicated by PASI (R = 0.23, p = 0.016) and BSA (R = 0.21, p = 0.023). Although obesity appeared to be associated with increased clinical severity, no significant correlation with DLQI was observed. [2].

Similarly, findings from a retrospective cohort study by Rompoti et al. support the notion that obesity (BMI ≥ 30 kg/m²) is a major comorbidity of psoriasis, serving as both a risk factor and a marker of increased disease severity [18]. These results align with those of Timis et al. Chi‑square testing indicated a significant association between obesity and psoriasis (p = 0.030) [17]. However, no significant correlations were found between PASI or DLQI scores and abdominal circumference, indicating that overall BMI, rather than fat distribution, may be the key factor influencing disease severity. 

Efficacy of Risankizumab in Patients with Moderate to Severe Plaque Psoriasis Based on BMI

Caldarola et al. reported the results of an analysis that showed that BMI did not have an impact on the treatment response [19]. Vaiopoulos et al. documented no major differences related to gender, disease duration, onset, or body weight [20]. Gargiulo et al. observed that BMI showed no significant impact week 52 onward in PASI measures [21]. Crowley et al. found that risankizumab led to higher PASI90 rates than secukinumab at week 52, regardless of BMI. Overall, BMI and other covariates did not significantly affect risankizumab effectiveness [6].

However Martorell-Calatayud et al. stated that risankizumab was more effective in patients with a BMI <30 kg/m², treatment-naïve patients, and those without psoriatic arthritis. While both groups had similar numbers achieving PASI100, non-obese patients led to earlier achievement of PASI ≤2 at week 2 [10].

Ibba et al. declared that patients without any cardiometabolic diseases had lower mean age, BMI, disease duration, and baseline PASI scores than those with at least one CMD. After one year of risankizumab treatment, PASI90 and PASI100 responses were achieved by 80.3% and 63% of patients with CMDs, compared to 83% and 63.2% of those without CMDs. Risankizumab might offer extra benefits for patients with cardiometabolic disease because it reduces inflammation beyond just improving skin symptoms [22].

In the study by Martorell-Calatayud et al., BMI was directly analyzed, showing that patients with BMI ≤ 30 kg/m² had a significantly better clinical response, especially at weeks 4, 16, and 52 where they more frequently achieved PASI ≤ 2. While PASI100 was achieved by 67% of patients overall, the data suggest that obesity may reduce the optimal response to risankizumab [10].

In contrast, Tejedor et al. did not stratify outcomes by BMI, but included a population with a high prevalence of comorbidities such as psoriatic arthritis and previous biologic use. Despite this, clinical outcomes remained strong at 64 weeks, with 71.4% reaching PASI75, 61.9% PASI90, and 57.1% PASI100. These findings suggest that risankizumab may maintain its efficacy even in patients with metabolic compromise [23].

While BMI may influence response magnitude, as shown by Martorell-Calatayud et al. [10], the overall evidence from Papp et al., Ibba et al., and Tejedor et al. suggests that risankizumab remains effective, safe, and durable across various patient profiles, including those with elevated BMI [15,23,24]. 

In general, the reviewed studies support the primary objective of this work: evaluate the impact of obesity, specifically body mass index (BMI ≥ 30 kg/m²), on the efficacy and safety of risankizumab in adults with moderate to severe plaque psoriasis. While no major differences in treatment response were observed between obese and non-obese patients, certain studies did suggest a faster or more frequent response in individuals with lower BMI. This underscores the importance of considering metabolic factors when assessing therapeutic outcomes.

The results obtained from the use of risankizumab in patients with psoriasis show a significant improvement in disease severity, reflected in a substantial reduction in PASI scores. Compared to other therapeutic options, such as ustekinumab and placebo, risankizumab demonstrated superior efficacy, achieving a greater reduction in PASI. These findings position risankizumab as a promising treatment for moderate to severe psoriasis [6,9,25,26].

Among the key advantages of risankizumab, as observed in the reviewed studies, are its high safety profile, low incidence of serious adverse effects, and consistent therapeutic response across different follow-up periods. These characteristics indicate that risankizumab is not only effective in the short term but also provides long-lasting benefits [7].

The reviewed studies reported no significant adverse effects that would limit risankizumab’s long-term use, making it a suitable option for patients with comorbidities, including obesity. This combination of sustained efficacy and safety enhances its appeal as a preferred biologic treatment for severe psoriasis [8].

Several studies, including those by Gargiulo and Crowley, found no significant impact of BMI on clinical outcomes such as PASI90 and PASI100, suggesting that risankizumab remains effective regardless of body weight [6,16]. However, some evidence indicates a slightly better response in non-obese patients. For instance, Martorell-Calatayud et al. reported that individuals with a BMI <30 kg/m² achieved improved PASI scores earlier and more frequently reached PASI ≤2 at key time points [10]. 

*Discussion* 

The available evidence supports risankizumab (Skyrizi®) as a highly effective biologic therapy for adults with moderate to severe chronic plaque psoriasis, providing rapid, clinically meaningful, and durable improvements in disease severity and patient-reported outcomes [7,15]. By selectively inhibiting the p19 subunit of IL-23, risankizumab disrupts the IL-23/Th17 axis central to psoriatic inflammation and keratinocyte hyperproliferation, consistent with the high rates of skin clearance observed across randomized trials and real-world studies [1,8].

In comparative settings, risankizumab has demonstrated superior efficacy versus other biologics, achieving higher proportions of patients reaching stringent endpoints such as PASI90 and PASI100 at clinically relevant timepoints, while also improving quality of life as reflected by DLQI responses [6].

Long-term follow-up indicates that these responses can be maintained for several years under continuous treatment [15], whereas treatment withdrawal is associated with higher relapse rates and a marked decline in PASI responses, underscoring the importance of maintenance therapy in a chronic, relapsing condition [13].

Across the reviewed studies, risankizumab has maintained a favorable safety profile, with a low incidence of serious adverse events and no consistent safety signals that would preclude long-term use [9,15].

Given the high prevalence of obesity among individuals with psoriasis and its association with increased disease severity, the impact of BMI on treatment outcomes is clinically relevant [2]. Most studies suggest that risankizumab remains effective across BMI categories with sustained PASI improvements [18], while some data indicate that non-obese patients may achieve faster or more frequent early low-residual disease states [22].

Overall, the evidence supports risankizumab as a highly effective systemic option for eligible patients, including those with cardiometabolic comorbidities, capable of delivering sustained skin clearance and meaningful improvements in patient quality of life.

## Conclusions

From a collective standpoint, risankizumab appears to be an effective and generally well‑tolerated therapeutic option for patients with moderate to severe psoriasis, based on the available evidence. Across the studies reviewed, consistent improvements in disease severity, reflected by PASI reductions, and a favorable safety profile were observed, including in populations with relevant comorbidities such as obesity.

However, it is important to emphasize that this review does not allow definitive conclusions regarding the impact of BMI on treatment outcomes. While several studies suggest comparable efficacy across BMI categories, others indicate a potential attenuation or delay in response among patients with higher BMI, particularly during early treatment phases. These findings are not uniform, and BMI was frequently analyzed as a secondary variable rather than a primary outcome.

Consequently, the role of BMI in modulating risankizumab efficacy remains uncertain. The heterogeneity of study designs, populations, follow‑up durations, and reporting of BMI‑specific data limits direct comparisons and precludes firm conclusions. As this is a scoping review intended to summarize existing evidence rather than establish causality or comparative effectiveness, the conclusions should be interpreted as suggestive rather than definitive.

Further well‑designed prospective studies, as well as systematic reviews and meta‑analyses specifically focused on overweight and obese populations, are required to clarify whether and to what extent BMI influences the therapeutic response and long‑term outcomes of risankizumab treatment.
